# Immunological Changes of Basophil Hyperreactivity to Sweat in Patients With Well-Controlled Atopic Dermatitis

**DOI:** 10.3389/fimmu.2022.883605

**Published:** 2022-06-29

**Authors:** Tomofumi Numata, Shunsuke Takahagi, Kaori Ishii, Satoshi Morioke, Takanobu Kan, Hayato Mizuno, Yuhki Yanase, Tomoko Kawaguchi, Akio Tanaka, Michihiro Hide

**Affiliations:** ^1^ Department of Dermatology, Graduate School of Biomedical & Health Sciences, Hiroshima University, Hiroshima, Japan; ^2^ Department of Pharmacotherapy, Graduate School of Biomedical and Health Sciences, Hiroshima University, Hiroshima, Japan

**Keywords:** atopic dermatitis, sweat antigen, histamine release, basophil, IgE, *Malassezia*, MGL_1304, dupilumab

## Abstract

**Background:**

Sweat aggravates atopic dermatitis (AD). In patients with AD, type-I hypersensitivity to sweat may be shown by histamine release from patients’ basophils in response to the semi-purified sweat antigen (QR), and the presence of specific immunoglobulin E (IgE) binding to MGL_1304, the component of QR. However, there has been no information on the immunological changes of type-I hypersensitivity to the sweat antigen in patients with well-controlled AD using topical corticosteroids (TCSs) and/or biologics as treatments.

**Method:**

Histamine-releasing tests using patients’ basophils and QR and the detection of serum IgE against MGL_1304 and mite allergen Der f 1 were performed in patients with AD who were well controlled by topical TCS with/without dupilumab for 53–96 weeks.

**Results:**

In total, 14 patients were enrolled. Seven patients received TCS therapy alone (TCS group), and seven patients received TCS with dupilumab therapy (dupilumab group). In all participants, the level of specific IgE against MGL_1304 decreased after treatments, but histamine release from basophils in response to QR did not show a statistically significant reduction; rather, it increased. In the dupilumab group, all changes in histamine release induced by QR (increase), the IgE level against MGL_1304 (decrease), and that against Der f 1 (decrease) were statistically significant, whereas the TCS group showed no significant change in any of them.

**Conclusion:**

The well-controlled condition for 53–96 weeks resulted in no reduction of the hyperreactivity of basophils against in patients with AD, even with the treatment with dupilumab. This study suggests persistent basophil hyperreactivity to sweat antigen over a year or longer.

## Introduction

Atopic dermatitis (AD) is a common chronic inflammatory skin disease, which is characterized by pruritic, eczematous lesions with the fluctuation of remission and relapse (1). It is often associated with high levels of serum immunoglobulin E (IgE) and a personal/family history of atopic diseases (1). The underlying pathogenesis of AD involves complex interactions among immune dysregulation, pruritus, and skin barrier dysfunction (2). The dysregulated immune system skews to a Th2 cell–mediated immune response, by which Th2 cytokines including interleukin (IL)-4 and 13 are activated in the lesions of AD (3). In addition to conventional treatments such as topical corticosteroids (TCSs), topical calcineurin inhibitors, and moisturizers, a monoclonal antibody against the alpha subunit of the IL-4 receptor, dupilumab, has recently emerged to inhibit both IL-4 and IL-13 signaling (4, 5). Its inhibition of the Th2 pathway improves AD skin lesions and skin barrier dysfunction, as well as IgE overproduction (5–7).

Sweat is one of aggravating factors for patients with AD (8, 9). In some patients with AD, type-I hypersensitivity to sweat is detected by the intradermal injection of autologous sweat and histamine release test (HRT) by using autologous sweat (9–11). The major antigen in sweat has been partially purified as the semi-purified sweat antigen (QR) (12). Recently, MGL_1304, a secreted protein by *Malassezia globosa*, has been identified as the histamine-releasing component in QR (13). Specific IgE binding to QR and MGL_1304 are detected in more than half of patients with AD (14). Furthermore, QR and MGL_1304 induce histamine release from basophils in 77% and 62% of patients with AD, respectively (12, 13). Although this large portion of patients with AD show basophil reactivity against QR, there is no information on the prognosis of type-I hypersensitivity to the sweat antigen. In this study, we performed an analysis of the change in basophil hyperreactivity and serum IgE titer against the sweat antigen in patients with AD that was well controlled by treatments with/without dupilumab.

## Materials and Methods

### Study Design

**Figure 1:**
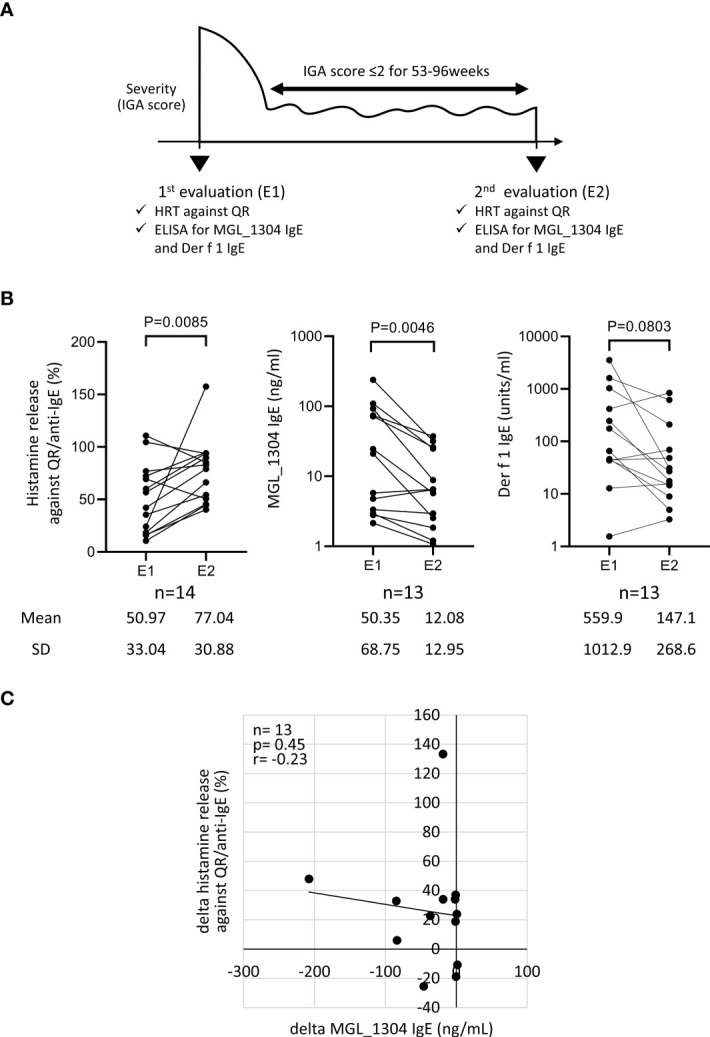
**(A)** Study design. **(B)** Histamine release from basophils in response to the semi-purified sweat antigen (QR) and levels of IgE to MGL_1304 and Der f 1 at E1 and E2 in total participants. **(C)** Correlation of the change of histamine release in response to QR to that of MGL_1304 IgE. Histamine release was expressed as (net % of histamine release induced by QR)/(net % of histamine release induced by anti-human IgE)×100 (%). “delta” means the changes of parameters calculated by the subtraction of values at E1 from those at E2. E1, the 1st evaluation; E2, the 2nd evaluation; HRT, histamine release test; IGA, Investigator’s Global Assessment.

The study protocol is shown in **Figure 1A**. Retrospective analysis for a total of 14 patients with AD was performed on the data obtained from electronic medical records at Hiroshima University Hospital. Participants were diagnosed as AD according to the Japanese guideline of the management of AD (1). They also had type-I hypersensitivity to sweat proven by HRT using QR (12). The type-I hypersensitivity to sweat was diagnosed in cases where anti-human IgE antibody (positive control) and QR induced the net histamine release of >10% and >5%, respectively (15). Two measurements of histamine release in response to QR, and serum IgE levels against MGL_1304 and mite allergen Der f 1 were performed at the first evaluation (E1) and the second one (E2). During the period from E1 to E2, the patients were treated with TCS or TCS with dupilumab but without any other systemic therapies for AD. The patients who entered the study had an Investigator’s Global Assessment (IGA) score of 2 or lower for 53–96 weeks, except for transient flares of AD within a month (**Table 1**). The IGA score at E1 was score 4 in five patients, 3 in four patients, 2 in three patients, and 1 in two patients, while the scores at E2 were score 2 in nine patients, 1 in three patients, and 0 in two patients. The change of the IGA score between E1 and E2 was 0 in three patients, −1 in four patients, −2 in six patients, and −3 in one other patient. All patients were advised to cope with sweat by taking a shower or wiping it with towels and to avoid dusty environments during the observation period. No uniform recommendation was given for patients to avoid other allergens during the study period unless the patient had an allergy to a specific antigen. The institutional review board of Hiroshima University Hospital approved the study protocol (approval number: E-2781, E-1716). Informed consent was obtained in the form of opt-out on the website.

**Table 1:** Patients’ characteristics.

Sex (male: female)		10:4	
Age, years (range)		44.1 ± 12.9 (21–66)	
Period between E1 and E2, weeks (range)		73.4 ± 16.1 (54–99)	
Period between E1 and the date of achieving IGA score ≤2, weeks (range)		2.0 ± 2.0 (0–6)	
Period between the date of achieving IGA score ≤2 and E2, weeks (range)		69.3 ± 16.3 (53–96)	
Treatments between E1 and E2		TCS, 7 TCS with dupilumab, 7	
Number of patients with skin diseases caused by *Malassezia*	Seborrheic dermatitis	0	
	Pityriasis versicolor	0	
	Malassezia folliculitis	3	
Number of patients sensitized to *Malassezia* species	*M. globosa*	14	
	*M. restricta*	14	
	*M. sympodialis*	13	
		<E1>	<E2>
Severity of AD (IGA score)	Score 0	None	2 patients
	Score 1	2 patients	3 patients
	Score 2	3 patients	9 patients
	Score 3	4 patients	None
	Score 4	5 patients	None
	Mean score	2.9 ± 1.1	1.5 ± 0.8
Serum IgE, ng/ml		12,169 ± 7,958	4,695 ± 4,928
TARC, pg/ml		4,913 ± 7,724	695 ± 552

Descriptive statistics are shown in mean ± standard deviation. E1, the 1st evaluation; E2, the 2nd evaluation; TCS, topical corticosteroids; IGA, Investigator’s Global Assessment; AD, atopic dermatitis; TARC, thymus- and activation-regulated chemokine.

### HRT Using the Semi-Purified Sweat Antigen, QR

QR was prepared by concanavalin A chromatography, anion-exchange chromatography, and reverse-phase chromatography in reference to histamine release activity from the basophils of patients with AD (12). HRT with basophils obtained from peripheral blood were performed as described previously (11, 16). Briefly, fresh blood was obtained with ethylenediaminetetraacetic acid (EDTA) from each donor by venipuncture. The whole blood was mixed with the same volume of 1% methylcellulose in saline and then allowed to stand at room temperature for 30 min. A supernatant with abundant leukocytes was collected, leaving red blood cells precipitated. After washing with the washing buffer, 136 mM sodium chloride/10 mM 4-(2-hydroxyethyl)-1-piperazineethanesulfonic acid (HEPES)/0.1% glucose/0.03% human serum albumin, the leukocyte fraction containing basophils was suspended in the reaction buffer, 136 mM sodium chloride/10 mM HEPES/0.1% glucose/0.03% human serum albumin/2 mM CaCl_2_/1 mM MgCl_2_. The suspended cells were stimulated with 0.33 μg/ml of goat anti-human IgE antibody (Bethyl Laboratories, Inc., TX, USA) or 10 ng/ml QR at 37°C for 40 min. After incubation, the cells and supernatant were separated by centrifugation and mixed with HClO_4_ to a final concentration of 2.5% to denature excess proteins. After centrifugation, the amounts of histamine extracted in the supernatant were measured using high-performance liquid chromatography (HPLC). The HPLC system consisted of an LC-20A (Shimadzu, Kyoto, Japan) using a VP-ODS column (Shimadzu, Kyoto, Japan) with an RF-10Axl fluorescence detector (Shimadzu, Kyoto, Japan) operating at an emission wavelength of 440 nm and an excitation wavelength of 360 nm. The percentage of histamine release was calculated using the ratio of the amount of histamine in the supernatant to the total amount of histamine in the supernatant and cells. For analysis, the value of histamine release against the sweat antigen was normalized by the maximum release of histamine induced by anti-IgE, namely, (net % of histamine release induced by QR)/(net % of histamine release induced by anti-human IgE)×100 (%).

### Enzyme-Linked Immunosorbent Assay (ELISA) for Specific IgE Against MGL_1304

A 96-well plate was coated with Smith2, a mouse monoclonal antibody against MGL_1304 (13), at 250 ng/well in phosphate-buffered saline (PBS) at 4°C overnight. After blocking, 10 ng/ml of the purified MGL_1304 from cultured *M. globosa* (13) were added in each well at room temperature for 2 h. After washing, the wells were incubated with several concentrations of a human monoclonal IgE against MGL_1304 (BioPorto Diagnostics A/S, Copenhagen, Denmark) (17), for standard reaction at 100 μl, and with 100 μl of patients’ sera diluted 1:10 with 10% blocking one solution (Nakalai Tesque, Inc., Tokyo, Japan) at room temperature for 2 h. After washing, each well was incubated with horseradish peroxidase (HRP)–conjugated goat anti-human IgE (Kirkegaard & Perry Laboratories, Inc., Gaithersburg, MD, USA). Color development was achieved by TMB substrate solution and stop solution (SeraCare Life science, Inc., MA, USA), and then absorbance at 450 nm was measured using a microplate spectrophotometer. A standard curve was constructed by plotting the concentrations of a series of standard reactions on the x-axis and absorbance on the y-axis. The concentration of MGL_1304 IgE was calculated in reference to the standard curve.

### ELISA for Specific IgE Against House Dust Mite Antigen, Der f 1

A 96-well plate was coated with 100 ng/well of Mite Extract-Df (LSL Co., Tokyo, Japan) in PBS at 4°C overnight. After blocking, the wells were incubated with 100 µl of patients’ sera diluted 1:10 with 10% blocking one solution at room temperature for 2 h. For a standard reaction in each experiment, the wells were also incubated with the serial dilution of the standard serum with high titer to Der f 1. The standard serum was obtained from a patient with AD sensitized to Der f 1. The subsequent method was the same as the ELISA for MGL_1304. The concentration of Der f 1 IgE was calculated in reference to the standard curve.

### Statistical Analysis

The changes of various parameters between E1 and E2 were calculated by the subtraction of values at E1 from those at E2. The statistical analysis was performed by GraphPad Prism (GraphPad Software, ver. 9.1, San Diego, CA, USA). A p-value of less than 0.05 was considered significant. Groups were compared by using the Wilcoxon matched-pairs signed rank test for paired samples. Correlation was tested with the Spearman’s rank test.

## Results

### Patients

A total of 14 patients with AD (men:women, 10:4) were recruited. Their clinical characteristics are shown in **Table 1**. The observation period from E1 to E2 was 73.4 ± 16.1 weeks (mean ± SD) including 69.3 ± 16.3 weeks in the well-controlled condition of IGA ≤2. During the observation period, seven patients received TCS therapy alone (TCS group), and seven patients received TCS with dupilumab therapy (dupilumab group). As for the sensitization to fungus, it has been reported that the sensitization to the sweat antigen, MGL_1304, is not correlated with *Candida albicans*, which belongs to another fungal genus (13). However, almost all participants were sensitized to more than one species of the *Malassezia* genus.

### Basophil Histamine Release in Response to QR and Serum IgE Binding to MGL_1304

The leukocyte fraction in blood was used to analyze histamine release from basophils in response to the sweat antigen. This fraction contained 0.48%–2.11% basophils, while the magnetic isolation of the basophil population achieved a basophil purity of 83.9%–93.6% by negative selection (n=4) (**Supplementary Figure 1A**, **Supplementary Materials and Methods**). The leukocyte fraction showed the same amount of histamine release in response to anti-IgE, QR, and MGL_1304 as the purified basophil fraction in patients with AD and hyperreactivity to QR (**Supplementary Figure 1B**, **Supplementary Material and Methods**). Thus, anti-IgE and the sweat antigen directly activate basophils regardless of the presence of other leukocytes in our HRT assay. The following experiments were performed with the leukocyte fraction containing basophils.

In the analysis of all participants, the level of IgE binding to MGL_1304 was significantly decreased at E2 from that at E1, but no significant reduction was observed in histamine release in response to QR and specific IgE binding to Der f 1 (**Figure 1B**) in the period between E1 and E2. No significant correlation was observed in the change of histamine release in response to QR and that of the level of IgE binding to MGL_1304 (*r=-0.23, p=0.45*) (**Figure 1C**). In this study, we calculated histamine release induced by QR with the normalization of maximum histamine release induced by anti-human IgE stimuli (positive control). However, non-normalized data showed the same tendency in the change of histamine release as normalized data (**Supplementary Figure 2A**). Non-normalized histamine release induced by QR was well correlated with histamine release induced by QR normalized with histamine release by anti-IgE (*r=0.74, p<0.0001*) at least for histamine release to QR (**Supplementary Figure 2B**).

### Stratified Analysis by Treatments With/Without Dupilumab

**Figure 2:**
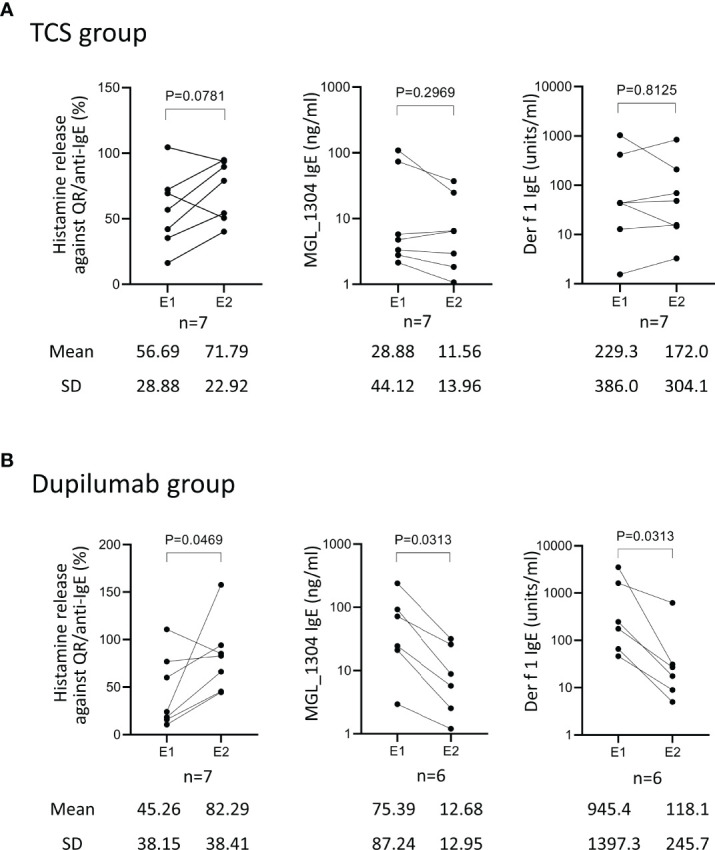
Histamine release from basophils in response to QR, and levels of IgE against MGL_1304 and Der f 1 at E1 and E2 in patients treated with TCS (TCS group) **(A)** and those treated with TCS plus dupilumab (dupilumab group) **(B)**. TCS, topical corticosteroid. Histamine release was expressed as (net % of histamine release induced by QR)/(net % of histamine release induced by anti-human IgE)×100 (%). E1, the 1st evaluation; E2, the 2nd evaluation.

Patients in the TCS group showed no significant difference between E1 and E2 in histamine release by QR and in the levels of IgE against MGL_1304 and Der f 1 (**Figure 2A**). In the dupilumab group, the levels of IgE against MGL_1304 and Der f 1 decreased, but histamine release in response to QR significantly increased at E2 from that at E1 (**Figure 2B**).

### Factors Affecting Histamine Release in Response to QR

**Figure 3:**
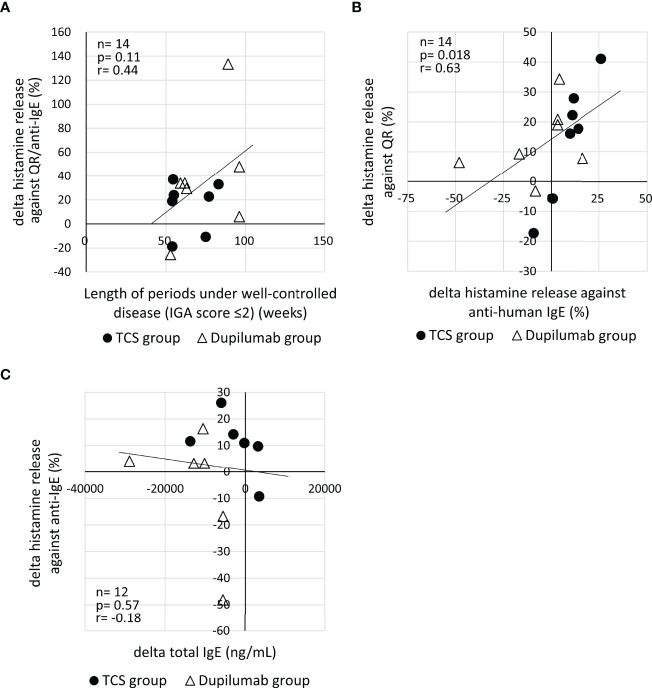
**(A)** Correlation of the change of histamine release in response to QR to the length of periods under a well-controlled disease (IGA ≤2). Histamine release was expressed as (net % of histamine release induced by QR)/(net % of histamine release induced by anti-IgE)×100 (%). **(B)** Correlation between the change of histamine release in response to QR and that of histamine release in response to anti-human IgE. **(C)** Correlation of the change of histamine release in response to anti-human IgE with the change of serum total IgE. “delta” means the changes of parameters calculated by the subtraction of values at E1 from those at E2. E1, the 1st evaluation; E2, the 2nd evaluation; IGA, Investigator’s Global Assessment.

To explore reasons why the hyperreactivity of basophils against QR showed no reduction in spite of the decrease of IgE against MGL_1304, we analyzed the relationship of histamine release in response to QR to the length of periods under a well-controlled disease (IGA ≤2), net histamine release in response to the anti-human IgE antibody, and total serum IgE level. There was no significant correlation of the change of histamine release in response to QR to the length of periods under a well-controlled disease (*r=0.44*, *p=0.11*) (**Figure 3A**). The change of net histamine release in response to QR and the change of net histamine release in response to anti-human IgE antibody were positively correlated (*r=0.63, p=0.018*) (**Figure 3B**). This correlation was significant in the TCS group (*r=0.89, p=0.012*) but not in the dupilumab group (*r=0.50, p=0.27*) (**Supplementary Figure 3A,B**). While most patients enrolled in this study showed an increase of histamine release in response to anti-human IgE and a decrease of total serum IgE at E2 compared to those at E1 (**Figure 3C**), there was no correlation between the change of histamine release against anti-human IgE and that of total IgE (*r=-0.18, p=0.57*).

## Discussion

In this study, we performed an investigation of the change of basophil hyperreactivity to sweat in patients who achieved a good control of AD. The histamine release activity of patients’ basophils in response to the semi-purified sweat antigen (QR) was not attenuated in the period of a well-controlled disease with the mean interval of 69 weeks (53–96 weeks) regardless of the use of dupilumab. Patients in the dupilumab group showed a significant reduction of specific IgE against MGL_1304 and Der f 1, whereas those in the TCS group did not. Thus, the suppression of the Th2 pathway by dupilumab reduced both the level of IgE against the sweat antigen and that against the mite antigen but did not result in a decrease of reactivity of basophils in response to the antigen.

The reduction of basophil hyperreactivity has not been observed in sweat allergy in this study but described in a type of food allergy. A previous study on the basophil hyperreactivity to hydrolyzed wheat (Glupearl 19S) sensitized by facial soap revealed a negative conversion of the basophil reactivity over the time of a year or longer by avoiding exposure to the antigen (18), suggesting that basophil hyperreactivity may decrease in food allergy developed by percutaneous sensitization. On the other hand, no significant reduction of histamine release in response to QR in this study implies persistent hyperreactivity of basophils to the sweat antigen in the period of 53–96 weeks. Although sweat allergy has likely developed by percutaneous sensitization, as was the case with Glupearl 19S allergy, the mechanism of sensitization to the sweat antigen in patients with AD may be somewhat different from that of food allergens. The avoidance of contact to food allergens on the skin is easier than that of the sweat antigen. Moreover, food antigens may induce tolerance *via* gastrointestinal uptake.

Several reasons may explain why the hyperreactivity of basophils against QR showed no reduction even in cases with the decrease of anti-MGL_1304 IgE in this study. First, MGL_1304 IgE levels might not have been sufficiently reduced to decrease the hyperreactivity of basophils. In mast cells, the crosslinking of only a few hundred molecules of the high-affinity IgE receptors are sufficient to induce mediator release (19). Second, the basophil function may have been impaired in the IgE receptor–mediated reaction at E1. It has been reported that severe dermatitis reduces the reactivity of basophils to IgE stimulation in patients with AD (20). In this study, AD lesions became well controlled between E1 and E2 in 9 patients, and the other 5 patients were maintained at a low disease activity (IGA ≤2) over the period. Ten of the total 14 patients showed higher levels of net histamine release from basophils in response to anti-human IgE at E2 compared to E1 (**Figure 3B**). Moreover, 9 of the 10 patients with increased histamine release in response to anti-human IgE also showed increased histamine release in response to QR (**Figure 3B**). This suggests that the maintenance of well-controlled disease activity may restore basophil reactivity *via* the high-affinity IgE receptor, resulting in the increase of histamine release in response to QR. The recovery of impaired basophil function during the time between two evaluations may explain a discrepancy between the change of antigen-specific IgE levels and that of histamine release in response to the sweat antigen. The recovery of basophil function may be partially associated with decrease of total serum IgE because most patients with decreased total IgE showed increased histamine release against anti-human IgE (**Figure 3C**). Further studies are necessary to confirm such possibilities of the basophil dysfunction using other parameters of basophil reactivity, including membrane activation markers and non-IgE receptor-mediated stimuli, such as complement component 5a (C5a) and formyl-methionyl-leucyl-phenylalanine (fMLP).

In this study, type-I hypersensitivity to sweat was assessed by HRT using QR but not evaluated by clinical aggravation due to perspiration. The *in vitro* detection of the hyperreactivity of basophils against the sweat antigen may not always be associated with clinical aggravation by perspiration. Nevertheless, prolonged hyperreactivity of patients’ basophils to the semi-purified sweat antigen shown in this study suggests that the extended management of sweat by showering (21, 22) and so on is necessary even after achieving an improvement of AD. The maintenance therapy to preserve a healthy skin barrier should also be taken to avoid flare due to the penetration of the sweat antigen through the damaged epidermis.

This study has a limitation in that a small number of patients were retrospectively involved in this study, and the observation period was only 53–96 weeks, despite many patients suffering from AD for a much longer period of time. Moreover, basophil reactivity was examined using the semi-purified sweat antigen, QR, but not MGL_1304, while the specific IgE was detected against MGL_1304. However, basophil reactivity in response to QR can be regarded as equivalent to that in response to MGL_1304 because MGL_1304 is considered to be identical to the histamine-releasing component in QR (see the reference 13 and **Supplementary Figure 1B**). Furthermore, the amount of MGL_1304 on the skin surface of individual patients was not quantified. Therefore, we cannot exclude the possibility that a difference between the amount of MGL_1304 on the skin surface at E1 and that at E2 may affect basophil reactivity to MGL_1304.

Collectively, this observation for 53–96 weeks showed no reduction of hyperreactivity of basophils against the sweat antigen in patients with AD that was clinically well controlled, even in cases treated with the Th2 pathway inhibitor, dupilumab. Further studies with longer observation periods are needed to determine the prognosis of hyperreactivity of basophils to sweat antigen.

## Data Availability Statement

The raw data supporting the conclusions of this article will be made available by the authors, without undue reservation.

## Ethics Statement

The study protocol was reviewed and approved by the institutional review board of Hiroshima University Hospital (approval number: E-2781, E-1716).

## Author Contributions

TN, ST and MH designed the study. TN, ST, MH, KI, YY, and ToK wrote the manuscript. KI, AT, HM, SM, YY, TaK and ToK contributed to data collection. TN and ST performed the statistical analysis and interpretation of the results. All authors read and approved the final manuscript.

## Funding

This study was supported by AMED under Grant Number JP19ek0410059 (ST and MH) and by JSPS KAKENHI under Grant Number JP21K08302 (ST), JP21K08346 (MH), and JP20K17319 (KI).

## Conflict of Interest

TN has received a research grant from Sun pharma and Kyowa-Kirin. ST has received a research grant from Sanofi, Maruho, and Taiho Pharma and an honorarium from Novartis. MH has received a research grant from GlaxoSmithKline, Kaken Pharmaceutical, Mitsubishi-Tanabe, Novartis, Sanofi, and Taiho Pharma, honorarium from Ely-Lilly, Kaken-Pharmaceutical, Kyowa-Kirin, Mitsubishi-Tanabe, MSD, Novartis, Sanofi, Taiho Pharma, Teikoku seiyaku, and Uriach. AT has received a research grant from Maruho, Sanofi, Eisai, and Mitsubishi Tanabe Pharma and an honorarium from Maruho, Torii Pharma, Mitsubishi Tanabe Pharma, Sanofi, and Taiho Pharma.

The remaining authors declare that the research was conducted in the absence of any commercial or financial relationships that could be construed as a potential conflict of interest.

## Publisher’s Note

All claims expressed in this article are solely those of the authors and do not necessarily represent those of their affiliated organizations, or those of the publisher, the editors and the reviewers. Any product that may be evaluated in this article, or claim that may be made by its manufacturer, is not guaranteed or endorsed by the publisher.

## Abbreviations

AD, atopic dermatitis; QR, semi-purified sweat antigen; E1, the 1st evaluation; E2, the 2nd evaluation; TCS, topical corticosteroid; IL, interleukin; HRT, histamine release test; *M.*, *Malassezia*; IGA, Investigator’s Global Assessment; HPLC, high-performance liquid chromatography; PBS, phosphate-buffered saline; HRP, horseradish peroxidase.
