# Supplementary material for: Immunological Changes of Basophil Hyperreactivity to Sweat in Patients With Well-Controlled Atopic Dermatitis
Source: Front Immunol. 2022 Jun 29;13:883605. doi: 10.3389/fimmu.2022.883605 (PMC9277351; doi:10.3389/fimmu.2022.883605)
Supplement: Supplementary file 1 [file Presentation_1.pdf]

Supplementary data

Materials and methods

Purification of basophils

Human peripheral blood mononuclear cells (PBMCs) were separated from the fresh blood of each donor with EDTA using Ficoll-Paque™ Plus (Cytiva Sweden AB, Upsala, Sweden) by density gradient separation. Basophils were isolated from PBMCs by magnetic depletion of non-basophils using the EasySep™ Human Basophil isolation Kit (STEMCELL Technologies Inc., Vancouver, Canada).

Analysis of the population of basophils by flow cytometry

The ratios of human basophils were analyzed in the leukocyte fraction and the purified basophil fraction by Attune Flow Cytometer (Thermo Fisher Scientific, MA, USA) using Allergenicity® kit (Beckmann Coulter, Brea, CA) according to the manufacturer's instructions.

Histamine release test (HRT) using the isolated basophils and the leukocyte fraction containing basophils

Both the isolated basophils and the leukocyte fraction were suspended in the reaction buffer, and stimulated with 0.33 µg/ml of goat anti-human IgE antibody (Bethyl Laboratories, Inc., TX, USA), 10 ng/ml the semi-purified sweat antigen; QR, or 10 ng/ml MGL_1304 at 37 degree C for 40 minutes, followed by the procedures mentioned in the body of the text.

Supplementary data

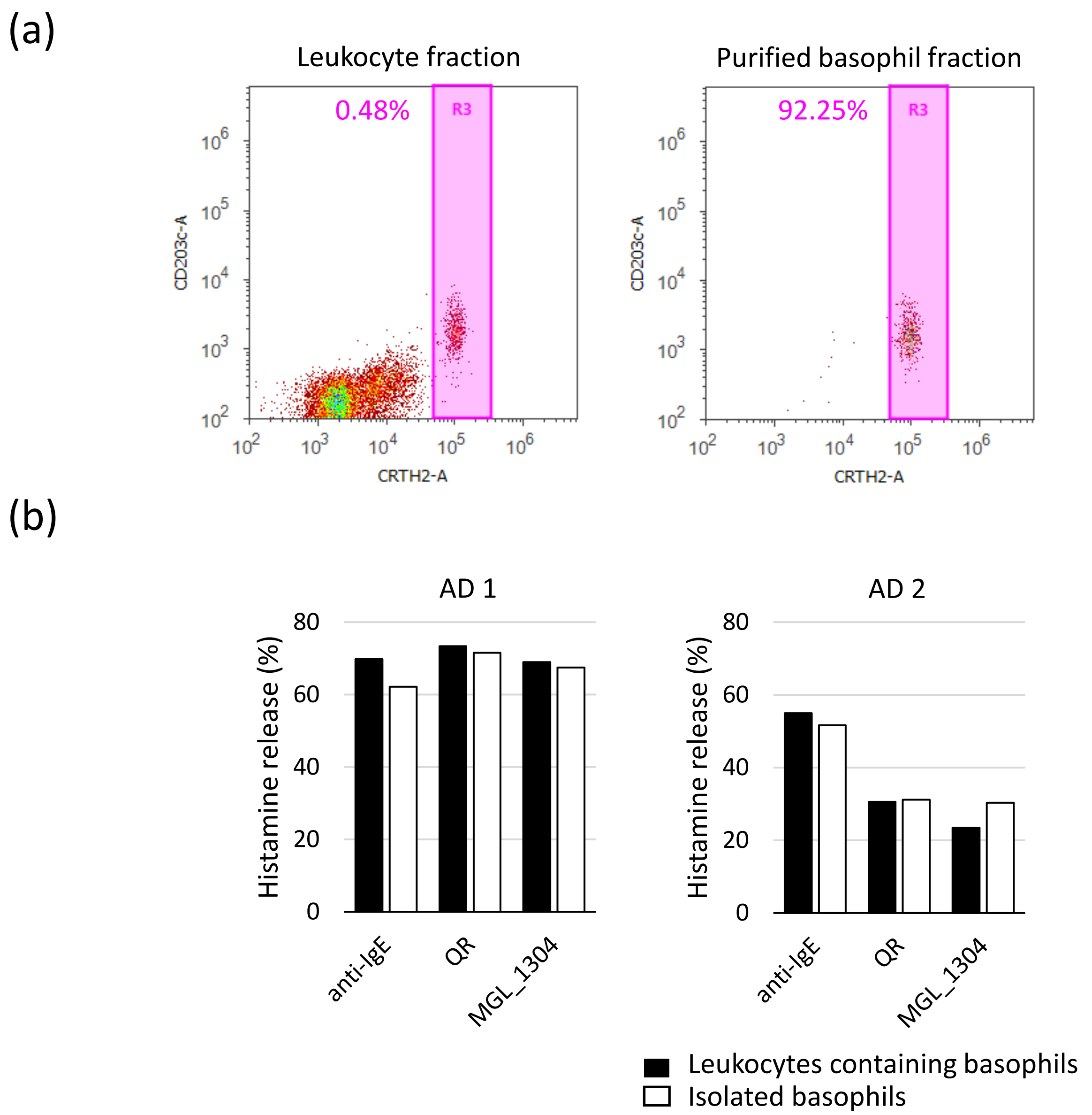

sFigure 1. (a) A representative data of flow cytometric analysis of basophils using the leukocyte fraction and the purified basophil fraction. Basophil population was gated in R3. (b) Histamine release using leukocytes containing basophils and isolated basophils. Samples from two patients with AD and hyperreactivity to QR were stimulated with anti-IgE, QR and MGL_1304. AD, atopic dermatitis.

Supplementary data

(a)

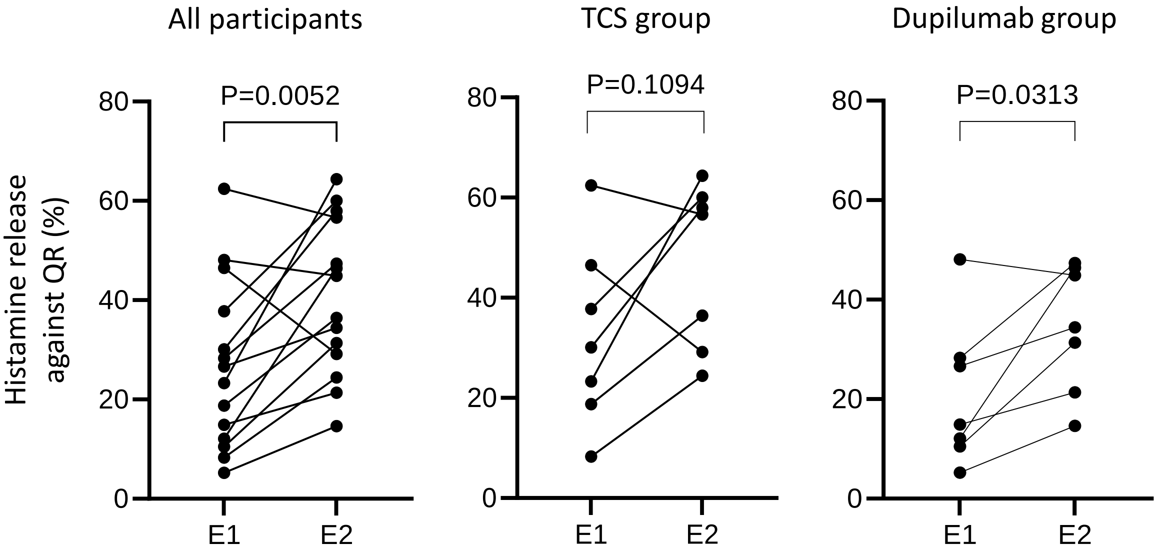

	n=14		n=7		n=7	
Mean	26.63	40.67	32.44	47.00	20.82	34.35
SD	16.98	15.63	18.18	16.45	14.68	12.87

(b)

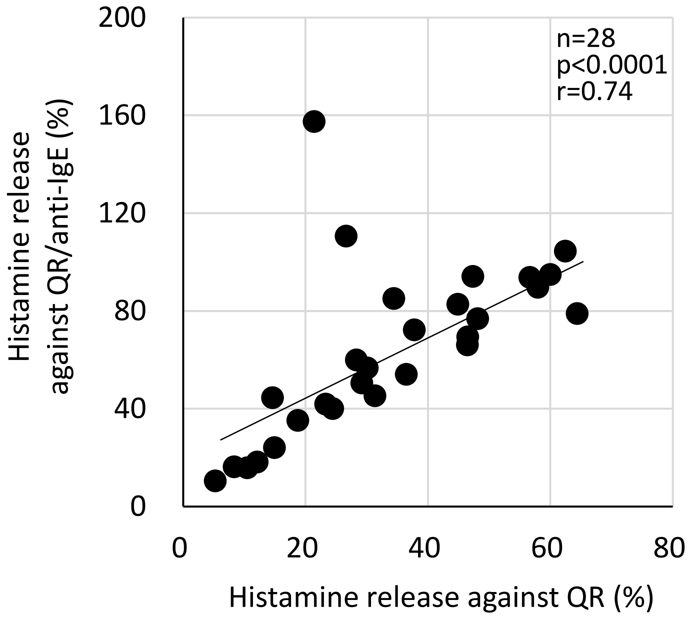

sFigure 2. (a) Non-normalized data of histamine release test using QR at E1 and E2 in total participants, and patients treated with topical corticosteroid alone (TCS group) or TCS plus dupilumab (Dupilumab group). The non-normalized data showed the same tendency in change of histamine release as those with normalization by histamine release induced by anti-IgE stimuli. (b) Correlation of normalized and non-normalized data of histamine release in response to QR. E1, the 1st evaluation; E2, the 2nd evaluation; TCS, topical corticosteroid.

Supplementary data

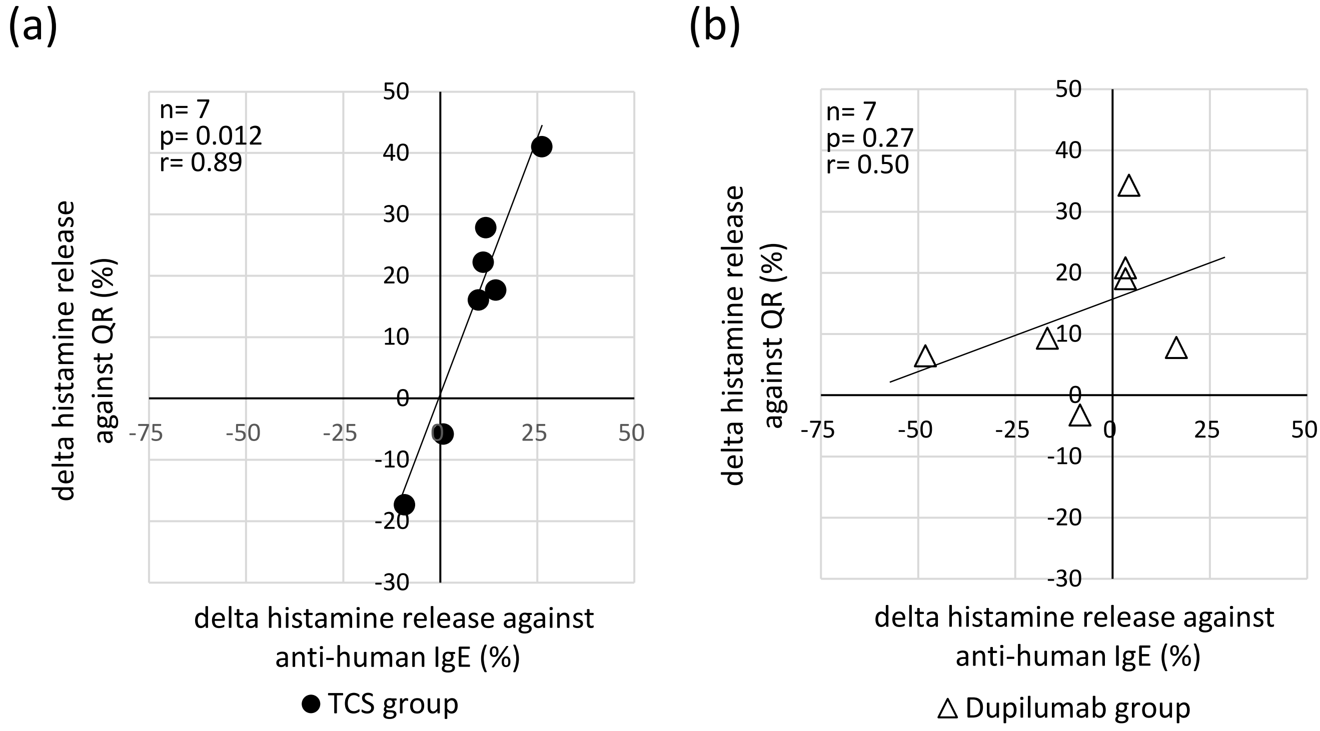

sFigure 3. Stratified analysis of correlation between the change of histamine release in response to QR and that of histamine release in response to anti-human IgE. Significant correlation was found in patients with topical corticosteroid alone (TCS group) (a), but not in those treated with TCS plus dupilumab (Dupilumab group) (b). “delta” means the changes of parameters calculated by the subtraction of values at E1 from those at E2. E1, the 1st evaluation; E2, the 2nd evaluation; TCS, topical corticosteroid.
